# Oncostatin M promotes STAT3 activation, VEGF production, and invasion in osteosarcoma cell lines

**DOI:** 10.1186/1471-2407-11-125

**Published:** 2011-04-11

**Authors:** Stacey L Fossey, Misty D Bear, William C Kisseberth, Michael Pennell, Cheryl A London

**Affiliations:** 1 Department of Veterinary Biosciences, The Ohio State University, Columbus, OH 43210, USA; 2 Department of Veterinary Clinical Sciences, The Ohio State University, Columbus, OH 43210, USA; 3 Comprehensive Cancer Center, The Ohio State University, Columbus, OH 43210, USA; 4 College of Public Health, The Ohio State University, Columbus, OH 43210, USA

## Abstract

**Background:**

We have previously demonstrated that both canine and human OSA cell lines, as well as 8 fresh canine OSA tumor samples, exhibit constitutive phosphorylation of STAT3, and that this correlates with enhanced expression of matrix metalloproteinase-2 (MMP2). While multiple signal transduction pathways can result in phosphorylation of STAT3, stimulation of the cytokine receptor gp130 through either IL-6 or Oncostatin M (OSM) is the most common mechanism through which STAT3 is activated. The purpose of this study was to evaluate the role of IL-6 and OSM stimulation on both canine and human OSA cell lines to begin to determine the role of these cytokines in the biology of OSA.

**Methods:**

RT-PCR and Western blotting were used to interrogate the consequences of OSM and IL-6 stimulation of OSA cell lines. OSA cells were stimulated with OSM and/or hepatocyte growth factor (HGF) and the effects on MMP2 activity (gel zymography), proliferation (CyQUANT), invasion (Matrigel transwell assay), and VEGF production (Western blotting, ELISA) were assessed. The small molecule STAT3 inhibitor LLL3 was used to investigate the impact of STAT3 inhibition following OSM stimulation of OSA cells.

**Results:**

Our data demonstrate that the OSM receptor (OSMR), but not IL-6 or its receptor, is expressed by all human and canine OSA cell lines and canine OSA tumor samples; additionally, OSM expression was noted in all tumor samples. Treatment of OSA cell lines with OSM induced phosphorylation of STAT3, Src, and JAK2. OSM stimulation also resulted in a dose dependent increase in MMP2 activity and VEGF expression that was markedly reduced following treatment with the small molecule STAT3 inhibitor LLL3. Lastly, OSM stimulation of OSA cell lines enhanced invasion through Matrigel, particularly in the presence of rhHGF. In contrast, both OSM and HGF stimulation of OSA cell lines did not alter their proliferative capacity.

**Conclusions:**

These data indicate OSM stimulation of human and canine OSA cells induces STAT3 activation, thereby enhancing the expression/activation of MMP2 and VEGF, ultimately promoting invasive behavior and tumor angiogenesis. As such, OSM and its receptor may represent a novel target for therapeutic intervention in OSA.

## Background

Osteosarcoma (OSA) is the most common malignant bone tumor in humans and dogs, although the incidence of disease in the dog population is approximately ten times higher than in people [1,2]. OSA in both species shares many features including the presence of microscopic metastatic disease at diagnosis, the development of chemotherapy resistant metastases, and dysregulation of several key cellular proteins including Met, ezrin and STAT3 [2-6]. Despite aggressive treatment including surgery and chemotherapy, little improvement in survival times has been achieved in either dogs or people over the past 15 years even with significant efforts directed at the incorporation of novel therapeutic approaches [7-9]. As such, the identification of key factors that regulate the aggressive biologic behavior of OSA, particularly with respect to metastasis, will be necessary if significant improvements in therapeutic outcome are to occur.

Oncostatin M (OSM) is a member of the IL-6 cytokine family produced by inflammatory cells and some tumor cells including primary human osteoblasts and the human OSA cell line MG-63 [10,11]. OSM stimulation of cells induces diverse functions across a variety of tissue types and cell lines such as modulation of growth and differentiation, inflammation, remodeling of extracellular matrix, and enhancement of metastatic capacity [11-14], however the exact role that this cytokine plays in bone biology has not yet been clearly defined [10,15].

OSM binds its receptor, oncostatin M receptor (OSMR), which exists as part of a heterodimer with the gp130 signal transducer, promoting reciprocal phosphorylation and activation of members of the Janus kinase family (JAK). Additionally, evidence suggests that OSM also acts through the leukemia inhibitory factor receptor (LIFR) and gp130 [16] with activation of DNA binding activity of STAT1, STAT3, and STAT5B [17]. Indeed, gp130 signaling cytokines such as OSM have been shown to be produced by mouse osteoblasts and osteocytes with differing effects through these receptors on osteoblast and osteoclast differentiation and activation [18-20]. Involvement of OSMR in bone biology was demonstrated by the osteopetrotic phenotype of OSMR- deficient mice [20]. The gp130 pathway has been shown to have multiple roles in bone growth, resorption, and formation thus making signaling through this pathway an interesting new area of study in bone biology and carcinogenesis [18].

Following OSM binding to OSMR and gp130, JAK2 is phosphorylated, which in turn phosphorylates STAT3 permitting nuclear translocation and modulation of gene expression [11,21,22]. Several transcriptional targets of STAT3 are important contributors to tumor biology and activation of STAT3 by gp130-mediated mechanisms is known to be oncogenic [23]. STAT3 has been implicated as being a central regulator of tumor progression through its transcriptional upregulation of VEGF, Mcl-1, and survivin, among others [24,25]. Additionally, members of the Src family of tyrosine kinases have been shown to be associated with and be activated through cytokine binding to gp130 in cancer cells [26,27]. Our previous work demonstrated that inhibition of STAT3 function in OSA cell lines using small molecule inhibitors downregulated MMP2 and VEGF expression and induced apoptosis suggesting that STAT3 activation may be an important regulator of the aggressive biologic behavior of OSA [6]. In support of this notion, a recent study demonstrated that human OSA patients whose tumors express high levels of phospho-STAT3 had a worse prognosis [28,29]. Lastly, expression profiling of pediatric OSA revealed that tumors with a poorer prognosis were associated with greater expression of genes enhancing cell migration and remodeling, many of which are transcriptionally regulated by STAT3 [30]. As such, the purpose of the following study was to explore the impact of OSM and IL-6 stimulation on OSA cell lines to begin to assess the role of the gp130 signaling pathway in OSA cell biology.

## Methods

### Cell Lines and Reagents

Canine OSA cell lines, OSA 8 and 16 were provided by Dr. Jaime Modiano (University of Minnesota, Minneapolis, MN) [6]. The canine D17 OSA cell line and human OSA cell lines U2OS and SJSA were purchased from American Type Cell Culture Collection (ATCC). Cell line authentication of human OSA cell lines SJSA and U2OS was recently completed by The Ohio State University Comprehensive Cancer Center Molecular Cytogenetics Shared Resource through karyotype analysis and comparison to that of the cell lines at ATCC. The canine lines and human line SJSA were maintained in RPMI-1640 supplemented with 10% fetal bovine serum, non-essential amino acids, sodium pyruvate, penicillin, streptomycin, L-glutamine, and HEPES (4-(2-hydroxethyl)-1-piperazineethanesulfonic acid) at 35°C, supplemented with 5% CO2. The U2OS cell line was cultured in McCoy's medium with 10% FBS and the same supplements as listed for the canine lines. The normal canine osteoblasts were obtained from Cell Applications (San Diego, CA) and maintained in Canine Osteoblast Growth Medium with 10% FBS. Human spleen total RNA was purchased from Ambion Biosystems (Austin, TX). The canine OSA tumor and normal spleen samples were obtained from dogs treated at The Ohio State University College of Veterinary Medical Center in compliance with established hospital policies regarding sample collection as part of the Biospecimen Repository. Collection procedures by the Biospecimen Repository are approved by the OSU IACUC (2010A0015, Canine Specimen Collection and Banking). Fresh tissue samples were immediately flash frozen in liquid nitrogen and stored in The Ohio State University College of Veterinary Medicine Biospecimen Repository. The tumors were evaluated and confirmed as OSA by board certified veterinary pathologists at The Ohio State University College of Veterinary Medicine.

### RT-PCR

RNA was extracted from untreated canine and human OSA cells and pulverized fresh frozen canine OSA tumor samples using TRIzol reagent (Invitrogen, Carlsbad, CA) according to the manufacturer's instructions. To generate cDNA, 2 μg of total RNA and the M-MLV reverse transcriptase kit (Invitrogen, Carlsbad, CA) were used according to the manufacturer's instructions. Next, 1/20 of the resultant cDNA was used for each PCR reaction in a total volume of 25 μl. Primers were designed and utilized for canine and human interleukin-6, interleukin-6 receptor, oncostatin M, oncostatin M receptor, gp130, and GAPDH (Table 1). Annealing temperatures for these reactions are listed in Table 1. All PCR products were run on a 2% agarose gel with ethidium bromide and visualized using the Alpha Imager system (Alpha Innotech Corp, San Leandro, CA).

**Table 1:** Primer pairs used for RT-PCR.

Gene	Primers (forward/reverse)	Annealing Temp (°C)
Canine IL-6	(5'-gagattccaaggatgatg/gcctctttgctgtcttcaca-3')	55
Canine IL-6R	(5'-gagatctgtgcagctcagcgactc/ctccactcacagaatacc-3')	55
Canine OSM	(5'-gtccttggactcctgttcctg/cactcagcatctccaagtc-3')	55
Canine OSMR	(5'-gtaaggtggaccgaggagatg/gagcaagccagtgtctctc-3')	55
Canine gp130	(5'-gaagcccagtccacc/ccttcatacaacgaatcc-3')	55
Canine GAPDH	(5'-accacagtccatgccatcac/tccaccaccctgttgctgta-3')	55
Human IL-6	(5'-gctgctcctggtgttgcctg/ggttgttttctgccagtg-3')	55
Human IL-6R	(5'-cagcagatgggctggcatgggaag/ccaagagcacagcctttgtc-3')	55
Human OSM	(5'-gtaccgcgtgctccttgg/cggcctcgccatctgcagc-3')	60
Human OSMR	(5'-gcaagtcaaggaaatgtcagtg/ccccaaggcagtgtccgtcc-3')	60
Human gp130	(5'-gaagcccaatccgccacataatttatc/cttcataggtgatcccacttg-3')	60
Human GAPDH	(5'-gcgcctggtcaccagggctgc/gaatttgccatgggtggaatc-3')	60

### Western Blotting

Protein lysates were prepared and quantified, separated by SDS-PAGE, and Western blotting was performed as described previously [6] on 2 × 10^6 ^OSA cells after stimulation with either PBS or recombinant human oncostatin M (rhOSM, 50 or 100 ng/mL; R&D Systems, Minneapolis, MN) or recombinant canine interleukin-6 (rcIL-6) (30 ng/mL; R&D Systems, Minneapolis, MN) for 0, 5, 10, or 30 minutes. Additionally, human OSA cell line SJSA was stimulated with either PBS, 50 or 100 ng/mL rhOSM, or 100 ng/mL rhOSM and the small molecule STAT3 inhibitor LLL3 [6] at 40 μM for 72 hours prior to collecting cells and preparing protein lysates that were separated by SDS-PAGE. The membranes were then incubated overnight with anti-p-STAT3 (Y705, Cell Signaling Technology, Danvers, MA), anti-p-JAK2 (Y1007/1008, Millipore, Temecula, CA), anti-VEGF (Santa Cruz Biotechnology, Santa Cruz, CA), or anti-p-Src (Y418, Invitrogen, Carlsbad, CA) after which they were incubated with appropriate horseradish peroxidase linked secondary antibodies, washed, and exposed to substrate (SuperSignal West Dura Extended Duration Substrate, Pierce, Rockford, IL). Blots were stripped, washed, and reprobed for β-actin (Santa Cruz Biotechnology, Santa Cruz, CA), total STAT3 (Cell Signaling Technology, Danvers, MA), total JAK2 (Cell Signaling Technology, Danvers, MA) or total Src (Cell Signaling Technology, Danvers, MA). Images shown are representative of all repeats of the experiments. Experiments were repeated twice.

### Immunoprecipitation

OSA cells (7 × 10^6^) cells were serum starved for two hours then treated with rhOSM (50 ng/mL) for 0 or 15 minutes. Cells were collected and lysate prepared as described previously [6]. The Rabbit TrueBlot™ kit (eBioscience, San Diego, CA) was utilized to immunoprecipitate canine gp130 using anti-gp130 antibody (Santa Cruz Biotechnology, Santa Cruz, CA) according to manufacturer's instructions. Protein was separated by SDS-PAGE and transferred to a PVDF membrane. Western blotting using an anti-Src or anti-STAT3 antibody (Cell Signaling Biotechnology, Danvers, MA) was performed after addition of the appropriate secondary antibody. The membrane was stripped and reprobed for gp130 and β-actin (Santa Cruz Biotechnology, Santa Cruz, CA).

### CyQUANT

OSA cells (2 × 10^3 ^cells/well) were seeded in 96-well plates overnight and incubated with PBS, 50, or 100 ng/mL rhOSM for 72 hours. Each treatment group was performed in four replicate wells. Prior to collection, media was removed and the plates were frozen at -80°C overnight before processing with the CyQUANT^® ^Cell Proliferation Assay Kit (Molecular Probes, Eugene, OR) according to manufacturer's instructions and analyzed as described previously [6].

### Gel Zymography

Cells were plated as previously described [6] and treated with PBS, 50, or 100 ng/mL rhOSM or 100 ng/mL rhOSM and the small molecule STAT3 inhibitor LLL3 40 μM [6]. Separate experiments were conducted with cells plated in a similar manner and treated with PBS, rhOSM (50 ng/mL), rhHGF (50 ng/mL), or the two together. Media was collected after 72 hours, processed, and gel zymography performed as described previously [6]. Images were scanned and analyzed using Image J.

### Invasion Assays

Canine (OSA8) and human (SJSA) OSA cells were plated in invasion assay experiments as described previously [31]. Briefly, cells were plated in the upper chamber in serum-free media with rhOSM (50 ng/mL) for all treatment groups. The lower chambers contained media with 10% fetal bovine serum alone (C10), C10 with rhOSM (50 ng/mL), C10 with rhHGF (50 ng/mL), or C10 with both cytokines at 50 ng/mL. Cells were incubated overnight to allow invasion through the Matrigel layer. Inserts were processed and cells counted as previously described [31]. Treatments were run in quadruplicate and cells from ten random fields from each replicate were counted.

### VEGF ELISA

125,000 canine (OSA8) or human (SJSA) OSA cells were plated in C10 media in a six well plate and cultured overnight. The media was removed and cells incubated for 24 hours in C1 media with PBS, OSM 50 or 100 ng/mL, or OSM 100 ng/mL + LLL3 40 μM. Media was removed and frozen at -80°C. VEGF expression was determined using the DuoSet ELISA Development System for canine or human VEGF (R&D Systems, Minneapolis, MN) according to manufacturer's instructions.

### Statistical Methods

In the invasion assays, we computed the average cell count per replicate and analyzed the means using a randomized block ANOVA (blocked on plate). Prior to analysis, the means were square root transformed in order to better satisfy the normality and equal variance assumptions of ANOVA. An overall F test of a difference in means across treatment groups was computed and pairwise comparisons of the groups were performed using Holm's method to control type-I error [32]. All experiments were performed two to three times. Statistical analysis of the VEGF ELISA data was performed using the Student's t-test. P values of less than or equal to 0.05 were considered statistically significant.

## Results

### Oncostatin M Receptor and gp130 are expressed in human and canine OSA cell lines

**Figure 1:**
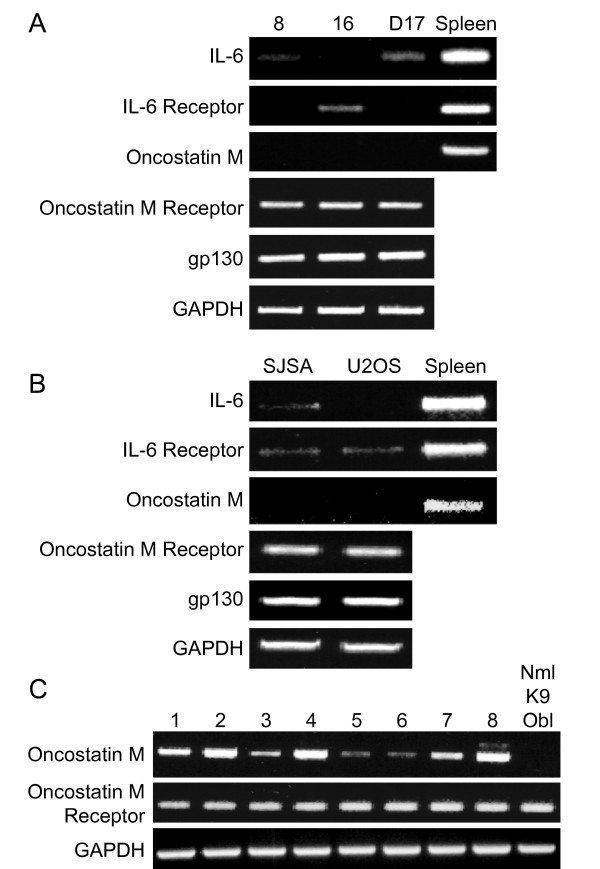
**Expression of OSM and IL-6 receptors in canine and human OSA**. RNA was collected from untreated **A) **canine (OSA8, 16, and D17) or **B) **human (SJSA and U2OS) OSA cell lines and RT-PCR was performed for IL-6, IL-6 receptor, OSM, OSMR, gp130, and GAPDH. **C) **RNA was collected from eight fresh frozen OSA tumors from canine patients and normal canine osteoblasts (Nml K9 Ob) and RT-PCR performed for OSM, OSMR, and GAPDH.

Expression of IL-6, IL-6 receptor, OSM, OSMR, and gp130 was determined in three canine (OSA8, 16, and D17) and two human (SJSA and U2OS) OSA cell lines by RT-PCR (Figures 1a and 1b, respectively). All cell lines expressed message for gp130 and OSMR; no expression of OSM was detected. IL-6 expression was variable and weak in canine OSA8 and D17 and human SJSA cells and IL-6 receptor was weakly expressed in canine OSA16 and human SJSA and U2OS cells. Given the apparent lack of IL-6/IL-6R expression in the OSA cells, we focused on OSM and its receptor in the fresh frozen OSA tumor samples from canine patients. OSMR expression was noted in all 8 canine tumor samples evaluated as well as the normal canine osteoblasts while OSM expression was detected in all samples although 2 of these were weak; normal canine osteoblasts did not express OSM. (Figure 1c).

### JAK2/STAT3 and Src phosphorylation is stimulated by Oncostatin M in OSA cell lines

**Figure 2:**
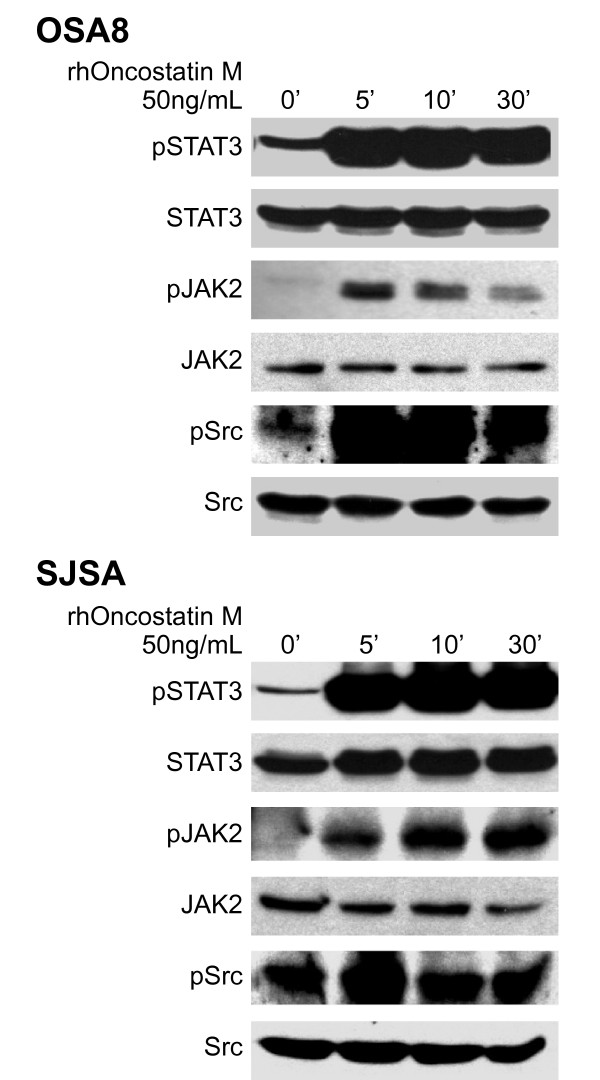
**Activation of STAT3, JAK2, and Src in OSA cell lines upon following stimulation with OSM**. Canine (OSA8) or human (SJSA) OSA cell lines were serum starved for two hours then stimulated with rhOSM (50 ng/mL) for 0 (no OSM stimulation), 5, 10, or 30 minutes. Protein lysates were generated and separated by SDS-PAGE and Western blotting for pSTAT3 (Y705), total STAT3, pJAK2 (Y1007/1008), total JAK2, pSrc (Y418), and total Src was performed.

OSM is known to activate the OSMR/gp130 heterodimer leading to phosphorylation of the JAK family kinases, in particular JAK2. Canine (OSA8) and human (SJSA) OSA cell lines were serum starved then stimulated with rhOSM (50 ng/mL) for 0, 5, 10, or 30 minutes before collecting cells for Western blotting (Figure 2). Basal levels of phosphorylated JAK2 were very low in both cell lines, however stimulation with OSM led to an immediate, transient increase in phosphorylation in OSA8 and a more sustained, time dependent increase in SJSA. Basal levels of STAT3 and Src phosphorylation were present as described previously in the OSA cell lines [6]; however, phosphorylation of both STAT3 and Src increased substantially within five minutes of OSM treatment. Levels of total protein for STAT3, Src, and JAK2 remained largely unchanged during all time points.

### JAK2/STAT3 phosphorylation is not stimulated by IL-6 in canine OSA

**Figure 3:**
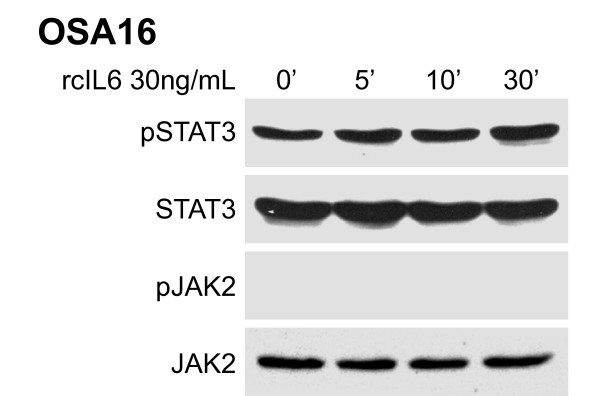
**Lack of IL-6 signaling in canine OSA**. The canine OSA cell line OSA16 was serum starved for two hours then stimulated with rcIL6 (30 ng/mL) for 0, 5, 10, or 30 minutes. Protein lysates were generated and separated by SDS-PAGE and Western blotting for pSTAT3 (Y705), total STAT3, pJAK2 (Y1007/1008), and total JAK2 was performed.

Given the expression of mRNA for IL-6 receptor in canine OSA cell line OSA16, we wanted to determine whether stimulation with its ligand IL-6 would impact JAK2 or STAT3 phosphorylation as had occurred with OSM. Cells were serum starved then treated with rcIL-6 for 0, 5, 10, or 30 minutes before cells were collected for Western blotting. JAK2 phosphorylation was not present at baseline and stimulation with IL-6 did not induce JAK2 phosphorylation (Figure 3). Basal STAT3 phosphorylation was present in OSA16 and this was not altered following IL-6 stimulation. Levels of total STAT3 and JAK2 proteins were not altered during all time points evaluated.

### Src and STAT3 are associated with gp130 in OSA cell lines with or without Oncostatin M stimulation

**Figure 4:**
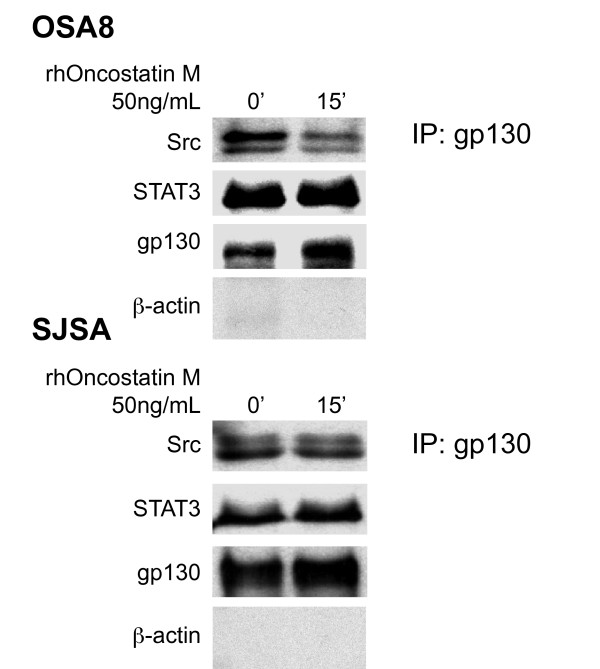
**Src and STAT3 are associated with gp130 in human and canine OSA cell lines**. Canine (OSA8) or human (SJSA) OSA cell lines were serum starved for two hours then left untreated or stimulated with rhOSM (50 ng/mL) for 15 minutes. Protein lysates were generated and immunoprecipitation performed for gp130. Co-precipitated proteins were separated by SDS-PAGE and Western blotting performed for total Src, total STAT3, gp130, or β-actin.

Binding of OSM to its receptor and gp130 results in recruitment of JAK2 to the receptor complex and subsequent recruitment and phosphorylation of STAT3. This association likely explains the activation observed in Figure 2, however the activation of Src after OSM binding is not as clear. Multiple members of the Src family of tyrosine kinases were coprecipitated with gp130 in lysates from multiple myeloma cells and stimulation with IL-6 led to increased activity of these Src family kinases [26]. We wanted to determine whether Src was associated with the gp130 complex in OSA cells as well. Canine (OSA8) and human (SJSA) OSA cell lines were serum starved for 2 hours then left untreated or treated for 15 minutes with rhOSM (50 ng/mL). Lysates were collected and gp130 was immunoprecipitated from the canine and human OSA cell lines (Figure 4). Western blotting revealed that Src and STAT3 were associated with gp130 in the presence or absence of OSM indicating that these proteins are part of the gp130 complex in these cell lines. The lack of β-actin in the co-precipitates confirmed the specificity of the immunoprecipitation experiment (Figure 4).

### Oncostatin M stimulation does not alter the proliferation of OSA cell lines

**Figure 5:**
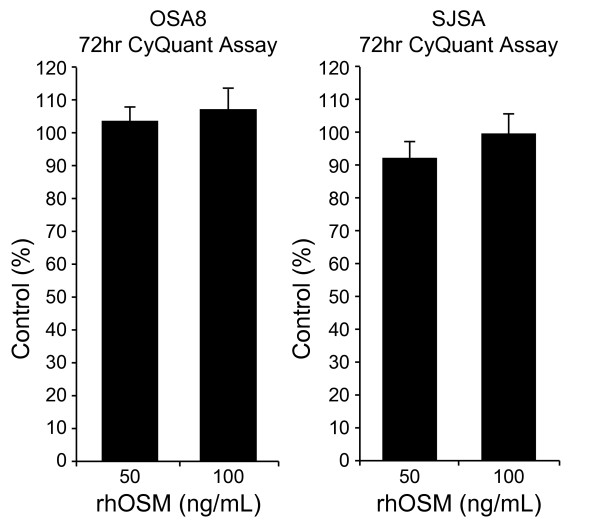
**OSM does not alter the proliferation of OSA cell lines**. Canine (OSA8) or human (SJSA) OSA cell lines were treated with 0, 50, or 100 ng/mL rhOSM for 72 hours in triplicate. Proliferation was analyzed using the CyQUANT cell proliferation assay kit. Proliferation values are listed as a percentage of PBS control and the bars represent the standard error of the mean.

OSM is a cytokine with multiple, divergent effects on cell proliferation differing among cell types and lines with growth inhibition effects reported in melanoma and glioma cells but stimulation of growth of Kaposi's sarcoma cells [11]. Canine (OSA8) and human (SJSA) OSA cell lines were incubated with 0, 50, or 100 ng/mL rhOSM for 72 hours and proliferation was assessed using the CyQUANT assay. As shown in Figure 5, there was no effect of OSM stimulation on OSA cell proliferation at either concentration.

### Oncostatin M stimulation of OSA cell lines enhances MMP2 and VEGF expression and tumor cell invasion

**Figure 6:**
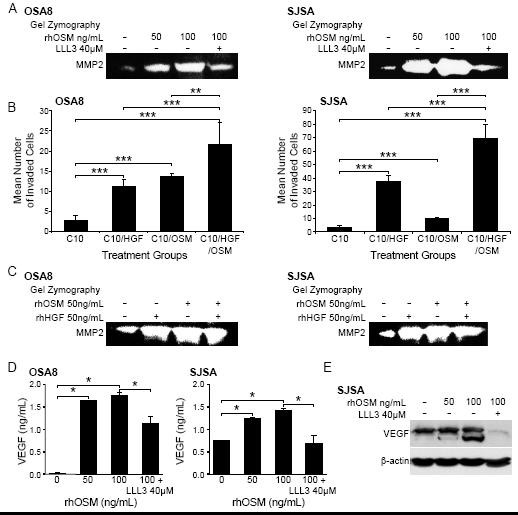
**OSM stimulation enhances MMP2 and VEGF activity and OSA cell invasion**. **A) **Canine (OSA8) or human (SJSA) OSA cells were treated with 0, 50, or 100 ng/mL rhOSM or 100 ng/mL OSM in combination with the small molecule STAT3 inhibitor LLL3 (40 μM) for 72 hours. Media was collected, processed, and gel zymography performed as described previously [6]. **B) **Canine (OSA8) and human (SJSA) OSA cells were plated in serum free media with 50 ng/mL rhOSM in the upper wells of plates for invasion assays. Cells were incubated overnight to allow for invasion through a layer of Matrigel. The lower chamber of each treatment group contained either 10% fetal bovine serum alone (C10), C10 media with rhOSM (50 ng/mL), C10 media with rhHGF (50 ng/mL), or C10 media with both cytokines at 50 ng/mL. Cells were counted in ten random fields in quadruplicate replicates. The bars refer to the standard error of the mean. **p < 0.01; ***p < 0.001 **C) **Canine (OSA8) or human (SJSA) OSA cells were treated with 0, 50 ng/mL rhOSM, 50 ng/mL rhHGF, or the two cytokines together at 50 ng/mL for 72 hours. Media was collected, processed, and gel zymography performed as described previously. **D) **Canine (OSA8) and human (SJSA) OSA cells were left untreated or incubated with 50 or 100 ng/mL rhOSM, or 100 ng/mL rhOSM + LLL3 40 μM for 24 hours. Media was collected and VEGF concentrations determined by ELISA. **E) **Human (SJSA) OSA cells left untreated or incubated with 50 or 100 ng/mL rhOSM, or 100 ng/mL rhOSM + 40 μM LLL3 for 72 hours. Protein lysates were generated and separated by SDS-PAGE and Western blotting for VEGF and β-actin was performed.

Previous work has shown that OSM promotes expression of MMPs including MMP1 and MMP3 in astrocytes [33], MMP1 and MMP9 in fibroblasts [33], and MMP1, MMP3, and MMP13 in chondrocytes [34]. Indeed, increased expression of MMP2 and MMP9 was linked to increased invasive capability in human and canine OSA [35,36]. We treated canine (OSA8) and human (SJSA) OSA cell lines with 0, 50, or 100 ng/mL rhOSM or 100 ng/mL OSM and 40 μM of the small molecule STAT3 inhibitor LLL3. We have shown in previous work that this STAT3 inhibitor down-regulates MMP2 expression at 72 hours following exposure [6]. OSM stimulation induced a dose dependent increase in MMP2 activity that was abrogated in the presence of LLL3 suggesting that the increase in MMP2 activity conferred by OSM stimulation is due in part to STAT3 activation (Figure 6a).

To determine whether the effect of OSM on MMP2 expression was biologically relevant with respect to tumor cell invasion, we cultured canine (OSA8) or human (SJSA) OSA cells in inserts containing serum-free media and rhOSM (50 ng/mL) overlying a Matrigel substrate. These inserts were placed in wells containing either media with 10% fetal bovine serum alone (C10), C10 with rhOSM (50 ng/mL), C10 with rhHGF (50 ng/mL), or C10 media with both cytokines together at same concentrations (50 ng/mL). After 18 hours of incubation, OSA cell lines treated with either cytokine alone exhibited significantly enhanced invasion as compared to media alone. (Figure 6b). Furthermore, invasion of OSA cells treated with both rhOSM and rhHGF was significantly greater than that observed with either cytokine/growth factor alone. Upregulation of MMP2 activity was observed following treatment with rhOSM alone, rhHGF alone and both OSM and HGF in combination (Figure 6c). Finally, stimulation of the human OSA cell line SJSA with OSM led to dose dependent increases in VEGF protein expression that was largely abrogated by concurrent treatment with the small molecule STAT3 inhibitor LLL3 (Figures 6d and 6e).

## Discussion

The link between inflammation and carcinogenesis is well known; experiments have implicated many components of the inflammatory cascade such as prostaglandin E2 and IL-6 as key players in tumor development, growth, and metastasis [37,38]. These inflammatory cytokines and growth factors, either generated by the tumor cells themselves in an autocrine manner or derived from inflammatory or stromal cells in the tumor microenvironment, have received much attention as potential targets for therapeutic intervention [37,39,40]. Indeed, these cytokines trigger the activation of many signaling pathways known to contribute to tumorigenesis and chemoresistance such as the JAK/STAT and Ras/Raf/MAPK pathways [11]. We had previously shown that STAT3 activation was present in a substantial number of OSA cell lines and primary canine OSA tumor samples and that inhibition of STAT3 using either a small molecule inhibitor or siRNA resulted in death of OSA cells *in vitro *[6]. The purpose of the following study was to identify possible drivers of the observed STAT3 activation.

Our data demonstrate that OSM, a member of the IL-6 subfamily of cytokines, and components of the OSM signaling pathway are expressed in OSA cell lines and tumor samples, and that activation of the JAK/STAT3 pathway with OSM stimulation leads to increased production of MMP2, VEGF, and enhanced tumor cell invasion. These results suggest that this pathway may be important *in vivo *for OSA cell metastasis by facilitating the process of invasion and angiogenesis. Interestingly, expression of IL-6 and IL-6R was either very low or absent in the OSA cells and the cells did not respond to stimulation with IL-6 indicating that this cytokine is likely not an important contributor to OSA pathobiology.

OSM is known to affect a variety of biological processes including cell growth and differentiation, hematopoiesis, and inflammation [11]. It has also been implicated as having a role in bone remodeling [20,41] in part through stimulating osteoblast differentiation and activation. OSM can be expressed in the bone marrow compartment [42] and is secreted from activated lymphocytes, monocytes, and neutrophils [11,43]. Interestingly, breast cancer cells have been demonstrated to stimulate neutrophils to produce the cytokine [43] and experiments have shown that OSM is produced by multiple human osteoblast-like cell lines including the OSA cell line MG-63 and mouse osteoblasts and osteocytes [10,20]. Co-expression of OSM and its receptor was noted in the fresh frozen tumor samples while only OSM receptor was identified in the cell lines. Based on these data, it is possible that the OSM found in the tumor specimens is derived from local inflammatory or stromal cells in the OSA tumor microenvironment independent of or, as demonstrated with the breast cancer cell lines, under the influence of the tumor cells.

OSM activates JAK2 and STAT3 upon binding to its receptor in many cells including murine, rat, and human osteoblastic cells and osteosarcoma cell lines [21,22]. However, the role of this cytokine pathway in OSA tumor cell survival and metastasis has not been fully explored. Upon stimulation with OSM, we demonstrated marked increases in JAK2, STAT3, and Src phosphorylation in canine and human OSA cell lines. This signaling enhanced the production of VEGF which is consistent with activation of STAT3, as it could be blocked by the small molecule STAT3 inhibitor LLL3 [6]. It has been shown that OSM stimulation enhances VEGF expression in adipocytes [44] and that OSM stimulates strong phospho-STAT3 (tyrosine 705) in normal and keloid fibroblasts [45]. Given that OSM is present in all canine patient tumor samples, it is plausible to infer that OSM in the tumor microenvironment *in vivo *likely enhances OSA basal Src and STAT3 activation and JAK2 phosphorylation. Indeed, the enhanced phosphorylation of Src and STAT3 and co-localization of Src and STAT3 with gp130 in the OSA cell lines following OSM stimulation suggest that a similar functional and spacial relationship exists between STAT3 and Src as shown by Schaeffer et al in multiple myeloma cells in which binding of IL-6 to gp130 led to activation of the Src family kinase Hck [26].

OSM is known to confer multiple, often divergent functions to various cell types including inhibition of melanoma and astroglioma tumor cell growth [43,46] and stimulation of proliferation of AIDS-related Kaposi's sarcoma cells and fibroblasts [47]. In OSA cells, OSM has been shown to downregulate osteoblast markers and induce glial fibrillary acidic protein [21], promote an osteocyte-like differentiation [12], and sensitize rat OSA cells to the antitumor effect of midostaurin [14]. However, our data indicate that treatment of canine and a human OSA cell lines does not impact their proliferation or viability. Other studies have shown that OSM has a role in regulating the MMPs as part of both wound healing and inflammation [11]. Enhanced MMP9 expression has been observed in astroglioma cell lines following OSM exposure [46] and breast cancer cells treated with OSM demonstrated increased VEGF production associated with detachment and invasion [43]. OSM stimulation has been linked to VEGF upregulation in normal adipocytes, liver, smooth muscle, and cardiac myocytes [44,48-50]. Lastly, OSM stimulation of astroglioma cells led to increased STAT3-dependent VEGF expression [51].

We observed increased MMP2 activity and VEGF expression with OSM stimulation of OSA cell lines that was partially abrogated by the small molecule STAT3 inhibitor, LLL3 [6]. Higher levels of VEGF expression in human OSA tumors have been shown to correlate with a significantly worse prognosis and the presence of lung metastasis [52,53]. Higher VEGF expression also has predictive value for survival of OSA patients [54]. With respect to canine OSA, one study found that pretreatment platelet-corrected serum VEGF levels correlated significantly with DFI in dogs with OSA following amputation and adjuvant chemotherapy [55]. Lastly, higher levels of plasma VEGF were found in more aggressive neoplasms in a survey of spontaneous canine tumors including those of the bone [56]. These data suggest that OSM stimulation of OSA cells may enhance VEGF production, thereby promoting angiogenesis, contributing to the metastatic cascade. Our data showed that OSM stimulation of OSA lines significantly enhanced the invasive behavior of OSA cells and that this was augmented in the presence of HGF. However, we have previously demonstrated that HGF stimulation of OSA cells does not promote STAT3 phosphorylation [31], and it is thus likely that HGF contributes to the observed invasion through mechanisms other than MMP2 production. As both OSM and HGF are likely to be relatively ubiquitous in the tumor microenvironment, it is possible they may work to promote early invasion and metastasis of OSA cells *in vivo*.

## Conclusions

Early microscopic metastasis is a frequent finding in OSA and the treatment of this disease will depend in part on identifying therapeutic targets to abrogate this process. We have shown in previous work that STAT3 dysregulation is frequently found in canine and human OSA cell lines and canine patient tumor samples. Our data here indicate that JAK2 and STAT3 are activated by the cytokine OSM and that this cytokine is present in canine patient tumor samples. Although OSM has various and at times contradictory functions in many tumor types, in our cell lines OSM enhanced MMP2 and VEGF expression and function in part through STAT3 activation, thereby promoting tumor cell invasion. These results support the notion that OSM may contribute to the invasive and metastatic nature of OSA through activation of STAT3, and as such may represent a viable pathway for therapeutic intervention.

## Competing interests

The authors declare that they have no competing interests.

## Authors' contributions

SF designed and carried out molecular experiments on OSA tissues and cell lines and drafted the manuscript. MB participated in RT-PCR design and performance and conducted VEGF ELISA experiments. MP conducted the statistical analysis of the invasion assays and assisted in experimental design. WK assisted in experimental design. CL conceived of the study, assisted in experimental design, and helped draft the manuscript. All authors read and approved the final manuscript.

## Pre-publication history

The pre-publication history for this paper can be accessed here:

http://www.biomedcentral.com/1471-2407/11/125/prepub
